# Supplementary material for: Discontinuous transcription of ribosomal DNA in human cells
Source: PLoS One. 2020 Mar 2;15(3):e0223030. doi: 10.1371/journal.pone.0223030 (PMC7051091; doi:10.1371/journal.pone.0223030)
Supplement: Attachment — Submitted filename: Reviewers.docx [file pone.0223030.s001.docx]

Michal Hetman

Academic Editor

PLOS ONE

December 4, 2019

Subject: Submission of revised paper PONE-D-19-25095

Dear Dr. Hetman,

Thank you for your email dated October 24, 2019 enclosing the reviewers’ comments.

We have revised the manuscript, addressing all critical remarks of the reviewers. In particular, we presented the results of new experiments with transfected cells and with incorporation of a different RNA nucleotide. The added or altered text is underlined in the revised version. Our specific replies to the reviewers are as follows (here the remarks requiring our reply are put in bold font):

***Reviewer #1****: The paper by Eugene Smirnov et al. describes the analysis of rDNA expression during about 2 h after release of human cells from cold treatment. FU incorporation was reduced by the cold treatment and the signal reached the normal level after 30 min of incubation at 37°C. Using transfected plasmid carrying Pol I gene fused with GFP or GFP-Fibrillarin construct they observed that cold treatment reduced the levels of Pol I in the FC/DFC units. In subsequent experiments they demonstrated that there are fluctuations of incorporated FU in the whole nucleoli during 120-150 min.The experimental data confirm the conclusion that rDNA genes are transcribed discontinuously during short period after incubation at 37°C.

The paper should be re-written.* ***Authors should first*** ***describe all experiments in the text in more detail. E.g., why they use this particular experiment in the text, why they use the transfections with Pol I gene and Fibrillarin gene.***

In the revised version of the text we furnished our description with further details. In particular, we observed that, since redistribution of pol I and fibrillarin in the cell nuclei is a common symptom of nucleolar pathology, the study of cells transfected with GFP-RPA43 or GFP-Fibrillarin was needed to assess the reaction of the cells to the chilling (Results, 1^st^ section, 3^rd^ paragraph).

***How they measure the intensity and amplitude. What are a.u. and a.e. units?***

As in our previous works, we measured the signal intensity in arbitrary units (a.u.), but “a.e.“ in Fig 7 was an error***,*** which we corrected in the revised text. “Amplitude” refers to the amplitude of the Fourier transform, i.e. the value of the periodogram. This was mentioned in the legend to the Fig 4 of our original manuscript.

***What secondary Ab were used in FU detection?***

We indicated the antibody in the revised Methods.

***Each panel in the Figure should be described.***

We extended and corrected the description of the Figures.

***Reviewer #2****: The work describes the study of discontinuous expression of rRNA genes. This manuscript builds on their previous work published in the Nucleus journal. In a new work, the authors investigated discontinuous expression of ribosomal genes after inhibition of transcription using cold stress. It seems that the data presented are not enough to convincingly illustrate the assumptions made.

1. The main emphasis in the work is made on the analysis of the intensity of label (FU) incorporation into the nucleoli of cultured cells. However,* ***the inclusion of FU depends not only on the intensity of expression, but also on the rate of its cellular uptake and phosphorylation in the cell. Therefore, confirmation of this key result by an independent method is necessary.*** ***The authors show in Fig. 2 and 3A (and in their previous work, this was also demonstrated) that the nucleolar accumulation of RPA 43 also changes with time. It is possible to confirm the presence of waves of intensity of ribosomal RNA expression using this method. The live cell imaging of RPA 43 during during the recovery of cells after cold stress (with estimations and statistical analysis) can illustrate described waves more accurately than the estimation of FU incorporation for overall cell population.***

We did the confirming experiments on the GFP-RPA43 transformed cells, as suggested by the reviewer. The results are described in the text (Results, section 4) and shown in the Fig 6 of the revised manuscript.  *2.* ***The authors indicate that the data on fluctuations and transcription cycles were obtained by averaging over several experiments (8 repetitions). It seems that it is necessary to present not only the result of averaging over 8 experiments, but also the curves for all individual experiments (plus, the result of averaging).***

In the revised manuscript we show the curves corresponding to individual experiments as well as the result of the averaging with the errors (Fig 4B,C and 5B,C of the revised manuscript).

***To appreciate how is the distribution of averaged values among the time points, instead of using bar graphs the data should be represented using box-plots with the individual values as dots.***

We created the box-plots, as suggested by the reviewer (Fig 4A and 5A of the revised manuscript).

***Also, the statistical analysis of detected fluorescence intensity fluctuations should be presented. These changes were statistically significant or not?***

We added statistical data (significance levels according to the Student’s t-criterion) showing significant change in the intensity of the FU signal and insignificant difference in the number of FC/DFC units between 15 min and 30 min after the cold treatment (see legend to the Fig 7)

*3.* ***I think that the experimental model used (restoration of transcription after cold stress) cannot be interpreted as synchronization. For human cells, this is very severe stress. And it seems that the subsequent processes should be interpreted as a process of recovery from stress.*** ***In this case, the transcription fluctuations can be connected with the process of cell restoration, which may differ from fluctuations in the control culture (which was described in the article in the Nucleus journal). Authors should at least briefly discuss such an interpretation of their data.***

We provide a brief discussion suggested by the reviewer (see Results, 1^st^ section, last paragraph and Discussion, 2^nd^ paragraph). There was indeed a recovery process, but it seemed to be limited to the first 30 min after the chilling, and it could not account for the regularly observed decrease of transcription intensity at 60 and 120 min (see Fig 4 and 5).

We would also observe that chilling does not seem to be a severe stress for the cultivated cells. Unlike the common methods of transcription inhibition (such as actinomycin, amanitin or DRB treatment), it causes no lasting abnormalities; in fact, we did not observe any changes in the nucleolar distribution of fibrillarin after the cold treatment (see Fig 2).

Relative mildness of the chilling shock may also be seen from the fact that cells are well preserved when kept outside the incubator (e.g. during transportation) at low temperature (without freezing).

***Reviewer #3****: The main goal of the study by Smirnov et al. “Discontinuous transcription of ribosomal DNA in human cells” was to evaluate nucleolar and nucleoplasmic transcription in two types of human cells (HeLa and epithelial limbal cells) after their synchronization with a low (4o C) temperature followed be the release from the cold shock as compared with untreated controls. Aims were reached by incubation of cells with 5-fluorouridine as precursor of RNA synthesis, expression of plasmids encoding RNA pol I subunit (RPA43) and fibrillarin fused with GFP. The intensity and number of signals were examined using a MatLab based software [ref 51] and Image J facilities. The authors concluded that: (1) chilling of cells results in arrest of pol I (nucleolar) and pol II (nucleoplasmic) transcription but does not displace all pol I complexes from their intrinsic locations; (2) cell release from the chilling conditions restores rDNA transcription to control values; (3) the restoration of rDNA transcription follows a wave-like manner (within 15-210 min of observation) and is discontinuous process.

Comments:* ***The major question concerns the principal novelty of the reviewed paper as compared with the papers recently published by the same authors in “Nucleus”*** *(M. Hornáček et al., Fluctuations of pol I and fibrillarin contents of the nucleoli. Nucleus, 2017, 8: 421-432; Smirnov et al., Discontinuous transcription Nucleus, 2018, 9: 149-160). In both publications, it is stated that ribosomal genes, like other genes, are transcribed in pulse-like manner (e.g., Hornáček et al., 2018, page 150), while it is well known that rRNA genes are transcribed during the entire cell cycle [ref. 49], which duration is much longer (roughly 24 hours) than the duration of observations in the current study (150-210 min).*

In the quoted work (Hornáček et al., 2017) we studied the fluctuations of pol I signal, but could not speak about transcription otherwise than hypothetically, since, for instance, our FRAP experiments indicated that most of the pol I molecules within FC/DFC units were not engaged in the current transcription. Only after developing the cold/release method of cell synchronization, we obtained the data related to the transcription directly. Hence, the straightforward title of our new study.

In the revised text we emphasized the novelty of the present work (Discussion, 2^nd^ paragraph).

*Minor questions:*

***Lines 25, 89 “in the populations of tumour derived” – Unclear meaning***

We changed “tumour derived“ to “HeLa“

***Line 92: Methods
Conditions for cell chilling should be described in this section instead of Results.***

We added this description to the Methods, but left it also in the Results for the convenience of the reader

***Lines 159, 160 (Fig 1) – It is unclear, where are the nuclear boundaries,***

In the revised manuscript, we showed the outlines of the nuclei on the indicated Figure.

***and how the authors determined that “… FU is accumulated predominantly in the FC/DFC units of the nucleoli” without using any markers for FC/DFC?***

In the revised manuscript we provided the reference (Results, 1^st^ section, 1^st^ paragraph) to our previous publication (Smirnov et al, 2016), where we demonstrated colocalization of FU and pol I fluorescent signals, as well as correspondence of the latter to electron microscopic images of the FC/DFC units.

***Lines 187, 190 (legend for Figure 3): “bars” should apparently be replaced by “columns”.***

We made the correction.

***Fig.3A: What are the vertical bars: SEM or standard deviation (Ϭ).***

The error bars signified SEM. We indicated this in the legends of the revised manuscript.

***Fig. 3B: SEMs (or Ϭ) are not indicated.***

As the legend says, the Fig 3B represents the total numbers of the FC/DFC units in the five cells (unlike Fig 3A, which represents the average intensities). Thus there are no statistical errors in this case.

***Lines 193, 194:*** *It is remained unspecified how the authors* ***distinguish*** *between negative labeling of cells with 5-FU (5-fluorouridine) caused by* ***inhibition of rDNA transcription from non-penetration of the precursor in cells in cold conditions.*** ***In addition, before incorporation in nascent pre-rRNA FU must be bound to ATP and this process most likely is suppressed by a low temperature.*** ***By other words, 5-FU was unincorporated not because genes were not transcribed, but because the precursor was inaccessible to nascent RNAs upon cold conditions.***

In the revised version of the manuscript, we present the data of a new experiment, in which BrUTP was used instead of FU (See Results, 1^st^ section, Fig 1B and Methods for the experiment description). The results seemed to show convincingly that cold alone inhibited the transcription in our experiments.

***Fig. 4 (Lines 207-213). A – there are no SEM (or Ϭ) on the columns and therefore it is impossible to compare differences between various time-points statistically. The latter makes the authors statement about a fluctuating manner of rDNA transcription uncertain.***

***Fig. 5 (Lines 241-246): See comments to Fig. 4.
Fig. 6 (Lines 264-267): See comments to Fig. 4.***

Combining the suggestions of the reviewers 2 and 3, we provided the error bars, as well as the graphs corresponding to individual experiments and box-plots in the Figs 4,5.

***B – on the periodograms, the horizontal axis scale does not correspond to the relative graphs in Fig. 4A.***

Our error was in applying the same term “time period” to different variables. In the revised text, to distinguish between the real time of the experiment and the parameter of the periodogram, we changed the first to ”Time“ and the second to ”Period*.“*

***The images below Fig. 7 are not described (are they copies?).***

Indeed, the images appeared in the pdf version as the result of an error. ***Unfortunately, figures are not numbered that makes their identification complicated.***

We added the numbers
