# Supplementary material for: Discontinuous transcription of ribosomal DNA in human cells
Source: PLoS One. 2020 Mar 2;15(3):e0223030. doi: 10.1371/journal.pone.0223030 (PMC7051091; doi:10.1371/journal.pone.0223030)
Supplement: Attachment — Submitted filename: letter2.docx [file pone.0223030.s002.docx]

Michal Hetman

Academic Editor

PLOS ONE

January, 22. 2020

Subject: Submission of second revised paper PONE-D-19-25095

Dear Dr. Hetman,

We submit second revision of the manuscript, addressing all critical remarks of the reviewers. The added or altered text is underlined in the revised version. Our specific replies to the reviewers are as follows (here the remarks requiring our reply are put in bold font):

Reviewer #1:
**All my concerns were addressed.**

Reviewer #2: **Minor questions**
**1. Line 336. The usual p-value in t-test is <0.05.**

We presented the result in the form suggested by the reviewer.

2. **The data in Fig. 4A and 5A. Is each curve based on a measurement of 50 cells? Maybe then it is worth presenting for each point the data not only on the mean, but also the standard deviation?**

We showed the deviations on the indicated graphs

3. **The new figures are not embedded in the logic of the article. In the current version, the last panel with box-plot in fig. 4a and fig. 4b (+ last panel with box-plot in 5a and fig. 5b) duplicate each other.**

The figure 4B contains mean values with standard deviations, whereas Fig 4A shows the extreme values of the set, the median and two other quartiles. We think that both indicated figures are useful.

4. **It is not clear why the error bars for the control cells are not provided (Fig. 4B and Fig. 5B, right panels)?**

This was only one experiment. It served to show that the variations of the transcription intensity in the control (the noise) were far exceeded by the variations in the experiment.

Reviewer #3:

(1) **The Abstract remains almost unchanged. For example, it does not include mentioning the results of BrUTP experiments, which were included to the revised paper (lines 176-183). According to my opinion, these experiments are crucially important for interpretation of the FU-labeling data. The same is also true for the last paragraph of Introduction, where the authors summarize the main paper results.**

We agree that these data are important, and we include them in the Abstract and Introduction of the second revised version.

.
(2) **Methods
It remains unclear, why the BrUTP labeling protocol is described only “briefly” (line 135). The publications cited by the authors for the scratch procedure used in the current study [55, 56] apply cell labeling with DNA replication but not with any transcription precursors.**

Applying the scratch method to the labelling of transcription we used BrUTP instead of DNA precursors. Otherwise, the procedure was the same as in the replication labelling. We clarify this point in the revised text.

**What was the duration of cell fixation with methanol (line 133)?**

The cells were fixed for 30 min. We provide this information in the revised text

**What was the working concentration of BrUTP?**

20µg/ml. We provide this information in the revised text.

**Line 140: “cy3-conjugated” should be replaced by “Cy3-conjugated”.**

We make this correction in the revised text.

With best regards,

Evgeny Smirnov
